# Retraction Note: Factors associated with decision-making power on family planning utilization among HIV-positive women attending public health facilities in Eastern Ethiopia

**DOI:** 10.1186/s40834-023-00215-1

**Published:** 2023-01-13

**Authors:** Hiwot Dejene, Derara Girma, Leta Adugna, Bilisumamulifna Tefera

**Affiliations:** 1 Department of Public Health, College of Health Sciences, Salale University, Fitche, Ethiopia; 2 Department of Public Health, College of Health Sciences, Mettu University, Mettu, Ethiopia

**Retraction Note: Contracept Reprod Med 7, 9 (2022)**

**https://doi.org/10.1186/s40834-022-00175-y**

The Editor-in-Chief has retracted this article because it contains material that substantially overlaps with the following article [1]. Derara Girma does not agree to this retraction. Hiwot Dejene, Leta Adugna and Bilisumamulifna Tefera have not responded to any correspondence from the editor/publisher about this retraction.
